# Preservation of common rhythmic locomotor control despite weakened supraspinal regulation after stroke

**DOI:** 10.3389/fnint.2014.00095

**Published:** 2014-12-22

**Authors:** Taryn Klarner, Trevor S. Barss, Yao Sun, Chelsea Kaupp, E. Paul Zehr

**Affiliations:** ^1^Exercise Science, Physical and Health Education, University of VictoriaVictoria, BC, Canada; ^2^Centre for Biomedical Research, University of VictoriaVictoria, BC, Canada; ^3^International Collaboration on Repair DiscoveriesVancouver, BC, Canada; ^4^Division of Medical Sciences, University of VictoriaVictoria, BC, Canada

**Keywords:** cutaneous reflex, supraspinal input, interlimb, afferent feedback, central pattern generator, rhythmic movements, rehabilitation

## Abstract

The basic pattern of arm and leg movement during rhythmic locomotor tasks is supported by common central neural control from spinal and supraspinal centers in neurologically intact participants. The purpose of this study was to test the hypothesis that following a cerebrovascular accident, shared systems from interlimb cutaneous networks facilitating arm and leg coordination persist across locomotor tasks. Twelve stroke participants (>6 months post CVA) performed arm and leg (A&L) cycling using a stationary ergometer and walking on a motorized treadmill. In both tasks cutaneous reflexes were evoked via surface stimulation of the nerves innervating the dorsum of the hand (superficial radial; SR) and foot (superficial peroneal; SP) of the less affected limbs. Electromyographic (EMG) activity from the tibialis anterior, soleus, flexor carpi radialis, and posterior deltoid were recorded bilaterally with surface electrodes. Full-wave rectified and filtered EMG data were separated into eight equal parts or phases and aligned to begin with maximum knee extension for both walking and A&L cycling. At each phase of movement, background EMG data were quantified as the peak normalized response for each participant and cutaneous reflexes were quantified as the average cumulative reflex over 150 ms following stimulation. In general, background EMG was similar between walking and A&L cycling, seen especially in the distal leg muscles. Cutaneous reflexes were evident and modified in the less and more affected limbs during walking and A&L cycling and similar modulation patterns were observed suggesting activity in related control networks between tasks. After a stroke common neural patterning from conserved subcortical regulation is seen supporting the notion of a common core in locomotor tasks involving arm and leg movement. This has translational implications for rehabilitation where A&L cycling could be usefully applied to improve walking function.

## Introduction

Supraspinal input, subcortical mechanisms and sensory feedback interact to coordinate limb movement during rhythmic locomotor tasks (Nielsen, 2003; Zehr and Duysens, 2004). Across different forms of rhythmic movement (e.g., swimming, walking, crawling, cycling etc.) similar coordination exists between these nervous system structures where common features of neural control facilitate the interactions between the arm and the legs (Dietz et al., 2001; Haridas and Zehr, 2003; Zehr, 2005). The relative contribution from various levels of control within the nervous system can be teased out with different experimental designs to determine which parts of the nervous system are important for controlling rhythmic movement. For example, volitional muscle activation (e.g., deliberate knee extension) reveals a shift toward strong supraspinal input whereas the same movement during a rhythmic task (e.g., knee extension during swing phase of walking) reveals a shift toward subcortical mechanisms (Zehr et al., 2007). Although different tasks rely more heavily on varying modes of control, all levels of the nervous system are required to fully support movement and are dynamically regulated.

This common nervous system control across rhythmic tasks can be determined by comparing the strength of connections during rhythmic activities probed during reflex studies. In neurologically intact (NI) participants these interactions can be seen in arm and leg muscles following a brief electrical pulse applied to a nerve in the hand or foot to evoke a reflex lasting at least 150 ms in the ongoing background electromyographic (EMG) activity. For example, cutaneous reflex amplitudes in arm and leg muscles were modulated in a similar way across tasks of level walking, incline walking, and stair climbing (Lamont and Zehr, 2006). Commonalities in control are also seen across walking, arm and leg (A&L) cycling and arm-assisted recumbent stepping, where similar phase-dependent modulation was observed despite differences in movement kinematics (Zehr et al., 2007). Factor analysis revealed that across these tasks, four principal components explained 93% of variance in background EMG and cutaneous reflex amplitude. Commonalities in cutaneous reflex modulation across different forms of rhythmic arm and leg locomotion reveal common central nervous system control (Zehr et al., 2007).

Given that the arms and the legs are functionally linked during locomotion and are subjected to similar nervous system control across rhythmic tasks, incorporating rhythmic arm movement in the rehabilitation of walking after stroke should be considered (Klimstra et al., 2009). Currently, rehabilitation is commonly provided with body-weight-supported treadmill training. However arm and leg cycling, which is similar to walking in terms of muscle activity, joint ranges of motion, and the neural pathways activated, might potentially strengthen interlimb connections in a similar way to walking (Zehr, 2005; Balter and Zehr, 2007). Therefore it would be useful to examine the extent of differences in neural control between A&L cycling and walking that may arise after stroke interrupts “normal” supraspinal regulation.

Following a stroke, decreased supraspinal input leads to alterations in muscle activation levels and patterns in locomotor tasks. Compared to NI participants, changes in burst durations, extent of co-contraction and amplitude modulations are observed during walking (Dimitrijevic and Nathan, 1970, 1973; Shiavi et al., 1987; Burridge et al., 2001; Zehr and Loadman, 2012). Deficits in the regulation of walking are due to interruption of connectivity between supraspinal and subcortical areas occurring as a result of the stroke lesion.

Despite differences in background EMG activity following stroke compared to NI participants, cutaneous pathways remain accessible and part of the “intact” regulation of sensory input still exits. For example, part of the stumble correction response, where stimulation to the top of the foot during the swing phase causes biceps femoris activation and tibialis anterior inhibition, normally observed in NI participants, was preserved in stroke participants (Zehr et al., 1998a). Interlimb connections have also been identified in stroke participants where cutaneous input can access reflex pathways in all four limbs, including the more affected (MA) limb, during rhythmic movement (Zehr and Loadman, 2012; Zehr et al., 2012). Interlimb reflexes were significantly phase-modulated and the depth of modulation for cutaneous reflexes was similar between stroke and NI participants (Zehr and Loadman, 2012).

The extent to which common neural regulation from supraspinal and spinal centers is conserved between locomotor tasks after stroke however, remains uncertain. Thus, the purpose of this study was to test the hypothesis that with decreased supraspinal input in chronic stroke, shared reflex systems from cutaneous networks remain viable and accessible across locomotor tasks. Since rhythmic arm and leg cycling and walking rely on contribution from subcortical circuits (Carroll et al., 2006), we hypothesized partial preservation of patterns of reflex modulation between the two tasks despite reduced supraspinal input after stroke. Background EMG and reflex modulation serve as proxies for the commonalities in neural function and a difference in these variables between tasks will be determined. The evoked responses for each participant were analyzed for the net reflex effect with the use of the average cumulative reflex EMG after 150 ms. This technique was employed because the major focus in this study is to determine the effect that reduced supraspinal regulation has on spinal cord and brainstem locomotor control centers (Zehr et al., 1998b; Komiyama et al., 2000). To probe arm and leg interactions, combined arm and leg stimulation was used as an index for arm and leg coupling where stimulation likely converges in shared reflex pathways (Nakajima et al., 2013).

## Materials and methods

### Participants

Twelve chronic stroke participants (≥6 months post infarct), between 58 and 80 years old, participated with written informed consent in a protocol approved by the Human Research Ethics Board at the University of Victoria.

### Experimental protocol

To examine similarities in rhythmic locomotor tasks, participants performed two tasks: (1) level walking on a motorized treadmill belt with 0% body weight support (Woodway Desmo M, Waukesha, WI, USA) and (2) seated arm and leg (A&L) cycling using a coupled arm and leg cycle ergometer (SciFit Pro II, Tulsa, Oklahoma, USA). Participants were instructed to maintain A&L cycling at 1 Hz and maintain waking at their self-selected walking speed.

### Electromyography

Electromyographic (EMG) recordings were made from tibialis anterior (TA), soleus (Sol), posterior deltoid (PD), and flexor carpi radialis (FCR) from both the more (contralateral; MA) and less affected (ipsilateral; LA) limbs. Skin was cleaned with alcohol and 1 cm surface EMG electrodes (Thought Technologies Ltd.) were applied in a bipolar configuration using a 2 cm inter-electrode distance over the muscles of interest. Grounding electrodes were placed over the patella and medial epicondyle of the elbow. EMG signals were pre-amplified 5000× and band-pass filtered at 100–300 Hz (P511 Grass Instrument, AstroMed, Inc.). Data were sampled at 1000 Hz (A/D converter; National Instrument, Austin, TX), and stored to a computer for off-line analysis.

### Nerve stimulation

In both tasks cutaneous reflexes were evoked via simultaneous stimulation of the nerves innervating the dorsum of the hand (superficial radial; SR) and foot (superficial peroneal; SP). Electrodes for SR nerve stimulation were placed just proximal to the radial head and for SP nerve stimulation on the ankle of the LA limbs. Appropriate stimulation location was checked by ensuring that radiating paresthesia was evoked into the appropriate cutaneous innervation areas of the SR and SP nerves. Cutaneous reflexes were applied with trains of 5 × 1.0 ms pulses at 300 Hz of isolated constant current stimulation (Grass S88 stimulator with SIU5 stimulus isolation and a CCU1 constant current unit AstroMed-Grass Inc., Canada). Stimulus intensity was set as multiples of the threshold for radiating paraesthesia (RT) at 2.2 × RT for the SR nerve, and 2.0 × RT for the SP nerve. Non-noxious stimulation intensities were found for each participant to ensure non-nociceptive pathways were stimulated. During both tasks, 120 stimulations were delivered pseudo-randomly with an inter-stimulus interval of 1–5 s.

### Movement timing

Timing events for arm and leg cycling were determined with custom-made optical encoders detecting position of the right arm crank throughout the movement cycle. Data were divided into cycles and aligned to begin with right arm top dead center. Walking cycle parameters (i.e., heel contact, toe-off) were obtained with the use of custom-made force sensors, located in the insole, and walking phases were divided to begin with LA heel strike.

**Figure 1:**
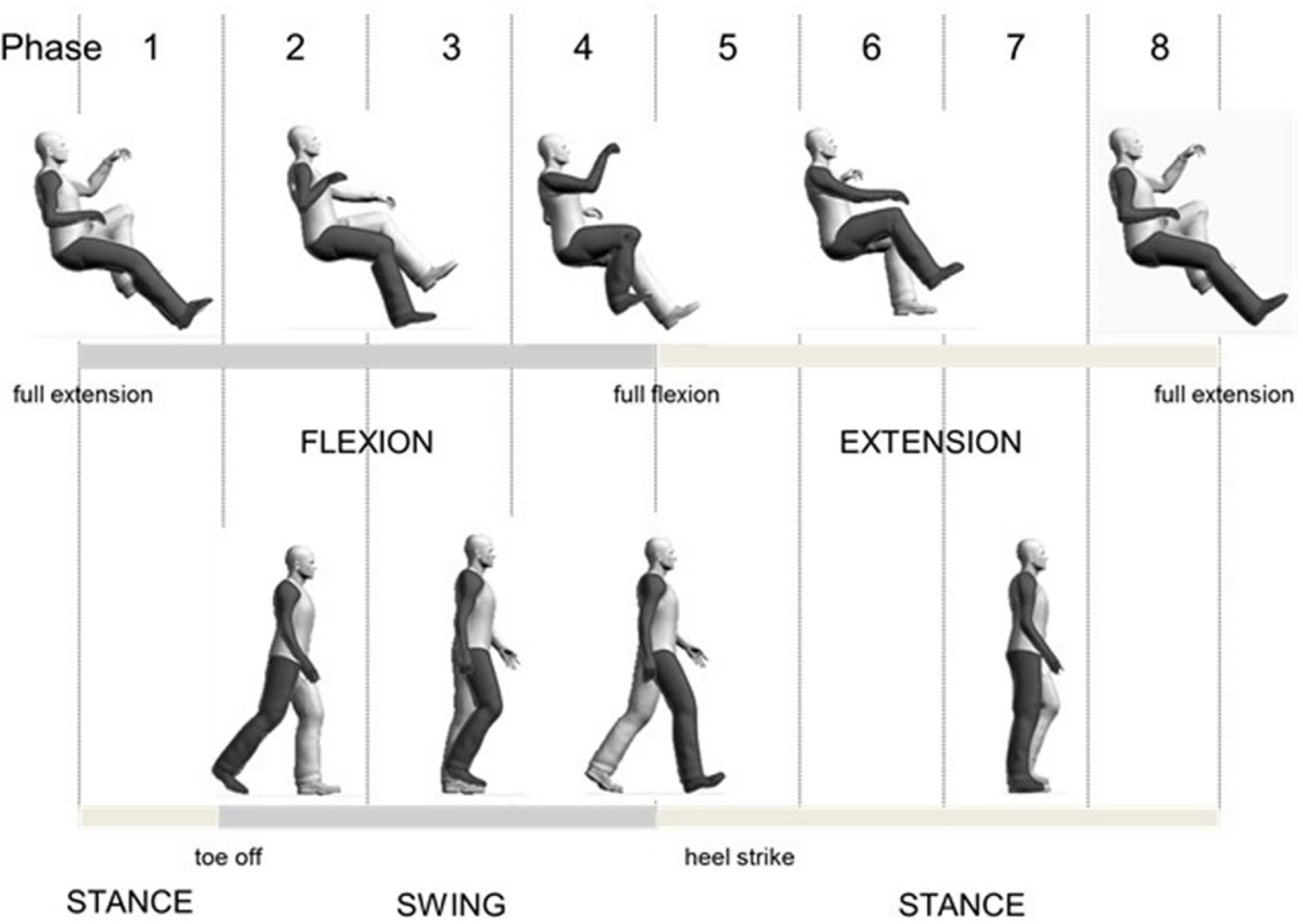
**Overall schematic diagram for relating arm and leg cycling to walking**.

For comparison of A&L cycling and walking, data were aligned to begin with maximum knee extension. A schematic diagram relating the phases of arm and leg movements for the tasks are shown in Figure 1. Eight equally divided phases are shown at the top and functional locomotor phases are compared below.

### Data analysis

EMG data were analyzed for background amplitudes and reflexes using custom-written software programs (MATLAB, The Mathworks, Inc., Natick, MA). Background EMG was obtained from steps without stimulation and was determined as the average response within a phase normalized to the peak response for each task for each participant. The average trace from the non-stimulated data was subtracted from the average trace of the stimulated data to produce a subtracted EMG “reflex” trace within each phase. Stimuli were then aligned to delivery within eight phases and within each phase, data were full-wave rectified, filtered, and averaged together. The stimulus artifact was removed from the subtracted reflex trace and data were then low-pass filtered at 30 Hz using a dual-pass, fourth order Butterworth filter.

Cutaneous reflexes were quantified as the average cumulative reflex over 150 ms following stimulation. This value is determined as the integral obtained at 150 ms divided by the time interval of integration to yield the overall reflex effect. If the value is positive, overall facilitation has occurred, if the value is negative, overall inhibition has occurred (Zehr et al., 1998b; Komiyama et al., 2000). This quantification method allows for interpretation of modulation of reflex pathways from spinal, brainstem and supraspinal centers where transcortical pathways have time to access and modify output from motoneurons during rhythmic activities and precedes any significant voluntary activation (Zehr et al., 1997). These values were normalized to the peak background EMG response for each task for each participant.

### Mathematical analysis

To examine basic patterns in neural control, a principal components analysis (PCA) was performed on background EMG and reflex data separately for all arm and leg muscles, recorded during A&L cycling and walking (Zehr et al., 2007) (MATLAB princomp function). From an 8 × 8 correlation matrix, showing linear dependence between muscles, eigenvalues were determined first. To increase loading on each principal component, an orthogonal varimax rotation of the eigenvalues was performed which grouped variables with similar activity together (Ivanenko et al., 2004). The percentage of the total variance explained by each principal component was simultaneously calculated (MATLAB pcacov function).

### Statistics

To compare between tasks, repeated measures analysis of variance (rmANOVA) was preformed separately for the variables of background EMG and net cutaneous reflex was used to determine significant differences (SPSS 18.0, Chicago, IL). The observed effect of each significant difference is also reported as the Cohen's effect size (d) where a small effect is *d* = 0.2, a medium effect is *d* = 0.5 and a large effect is *d* = 0.8 (Cohen, 2013). Cohen's d is useful for determining if any failure to observe significant differences was due to small sample sizes. Analyses were performed using the averaged normalized values for each subject. Using rmANOVA, differences in the pattern of response would be detected as a task-phase interaction indicating a difference in timing of peaks across phases between the two tasks. General amplitude differences in background EMG or net cutaneous reflex between tasks would be detected as a significant main effect of task. Any differences seen across phases, indicating phase dependent modulation of background EMG and net cutaneous reflex, would be seen as a significant main effect of phase. Taking a conservative approach and to examine all possible statistical differences, significant interaction and main effects tests were examined with paired samples *t*-tests to determine phase specific differences between tasks. Statistical significance was set at *p* ≤ 0.05.

## Results

### Background EMG

**Figure 2:**
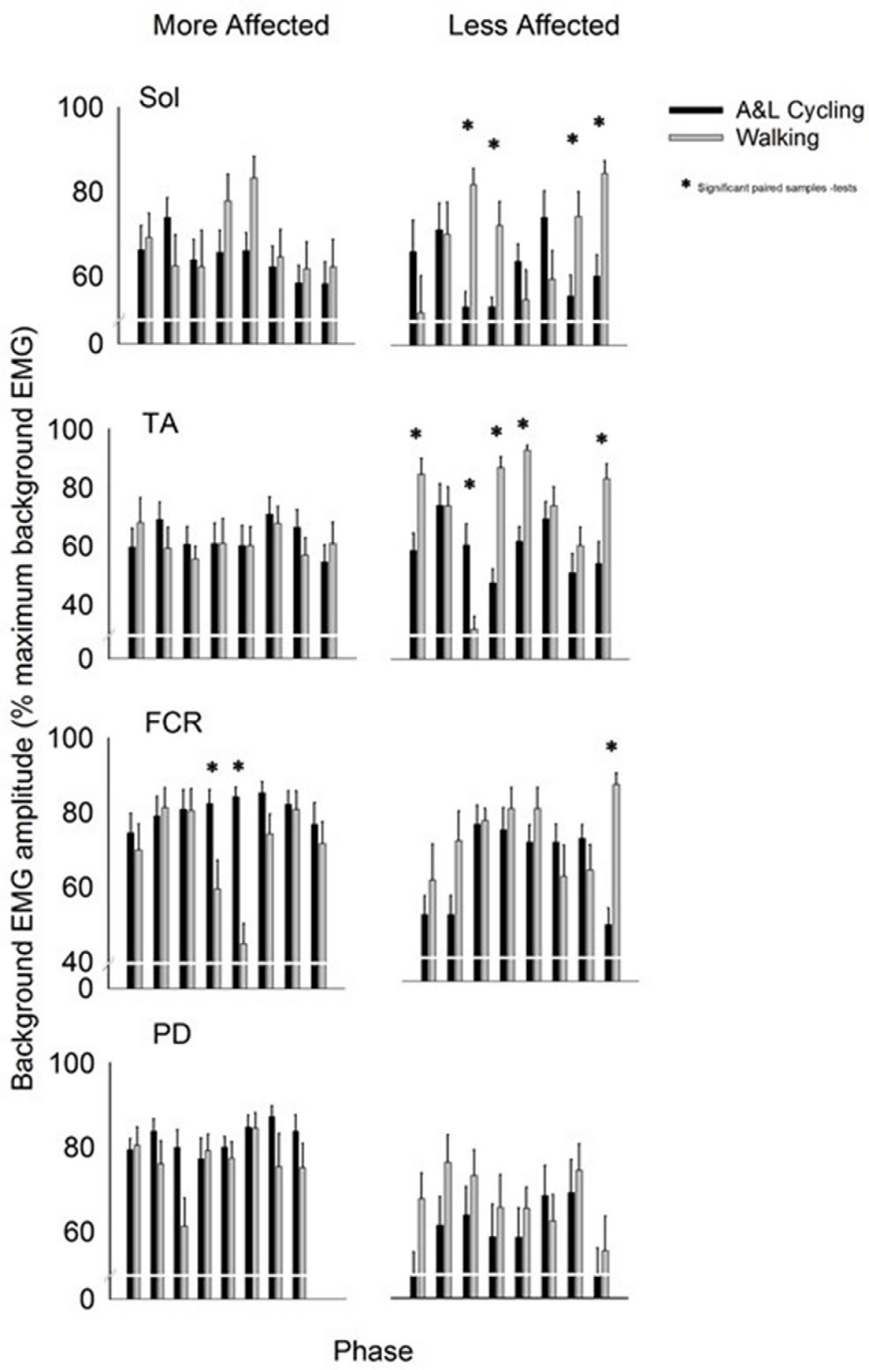
**Background EMG amplitudes for muscles of the more and less affected arm and leg averaged across all participants**. Black bars are for A&L cycling and gray bars are for walking tasks. The EMG amplitudes are means (± s.e.m.) from all participants and are normalized to the peak control (i.e., background) EMG recorded in each task. Significant difference between tasks (^*^) were calculated with a paired samples *t*-test. Abbreviations are: Sol, soleus; TA, tibialis anterior; FCR, flexor carpi radialis; and PD, posterior deltoid.

Background EMG patterns for the Sol, TA, FCR, and PD of the MA and LA limbs during SR and SP nerve stimulation for both A&L cycling and walking are shown as bar plots in Figure 2. Values for A&L cycling (black bars) and walking (gray bars) are normalized and expressed as percentages of the peak response for each task for each participant. Due to the varying capabilities of each stroke participant walking was maintained at 0.76 Hz and A&L cycling was maintained at 0.89 Hz and no significant differences (*p* = 0.549) in frequency were found between tasks. This allows for comparisons between tasks without the confounding effects of movement frequency and to match movement parameters in Zehr et al. (2007).

In the legs, there were differences in the pattern of background amplitude as differences in timing of the peaks, as indicated by a task-phase interaction for the LA Sol and LA TA [*F*_(7, 70)_ = 4.951, *p* < 0.000, *d* = 0.994 and *F*_(7, 70)_ = 9.211, *p* < 0.000, *d* = 0.999 respectively]. There was significant phase-dependent modulation for both tasks in LA TA {main effect of phase [*F*_(7, 70)_ = 7.519, *p* < 0.000, *d* = 0.997]}. In LA Sol and LA TA there was also a main effect of task [*F*_(1, 10)_ = 5.779, *p* = 0.037, *d* = 0.583 and *F*_(1, 10)_ = 15.456, *p* = 0.003, *d* = 0.942 respectively]. Some significant *post-hoc* differences, between A&L cycling and walking, were observed for LA Sol and LA TA and there were no significant differences for MA Sol and MA TA (see ^*^ in Figure 2). The small number of differences can be better appreciated by considering the number of phases in which significant differences could have been observed, which is 32 [equal to the number of phases (8) × number of muscle recorded (4)]. In this context, there were 9 differences out of 32 for SR+SP stimulation trials. These few statistically significant differences between tasks indicate that the extent of background EMG amplitude modulation was similar across tasks.

In the arms, there were few differences in the pattern of background amplitude as differences in timing of the peaks {task-phase interaction for only the MA FCR [*F*_(7, 70)_ = 4.036, *p* = 0.001, *d* = 0.977]}. There was significant phase-dependent modulation for both tasks in the MA FCR and LA FCR seen as a significant main effect of phase [*F*_(7, 70)_ = 3.507, *p* = 0.003, *d* = 0.954 and *F*_(7, 70)_ = 3.616, *p* = 0.002, *d* = 0.958 respectively]. Statistically significant differences between tasks were found in the MA FCR [*F*_(1, 10)_ = 13.941, *p* = 0.004, *d* = 0.919], LA FCR [*F*_(1, 10)_ = 6.909, *p* = 0.027, *d* = 0.649], and MA PD [*F*_(1, 10)_ = 7.382, *p* = 0.022, *d* = 0.688] but only a few significant *post-hoc* differences were apparent between A&L cycling and walking for the MA FCR and LA FCR (see ^*^ in Figure 2). When the number of phases with significant differences is considered, as described for the arm muscles above, there were 3 differences out of 32 for SR+SP stimulation trials.

### Reflex modulation

**Figure 3:**
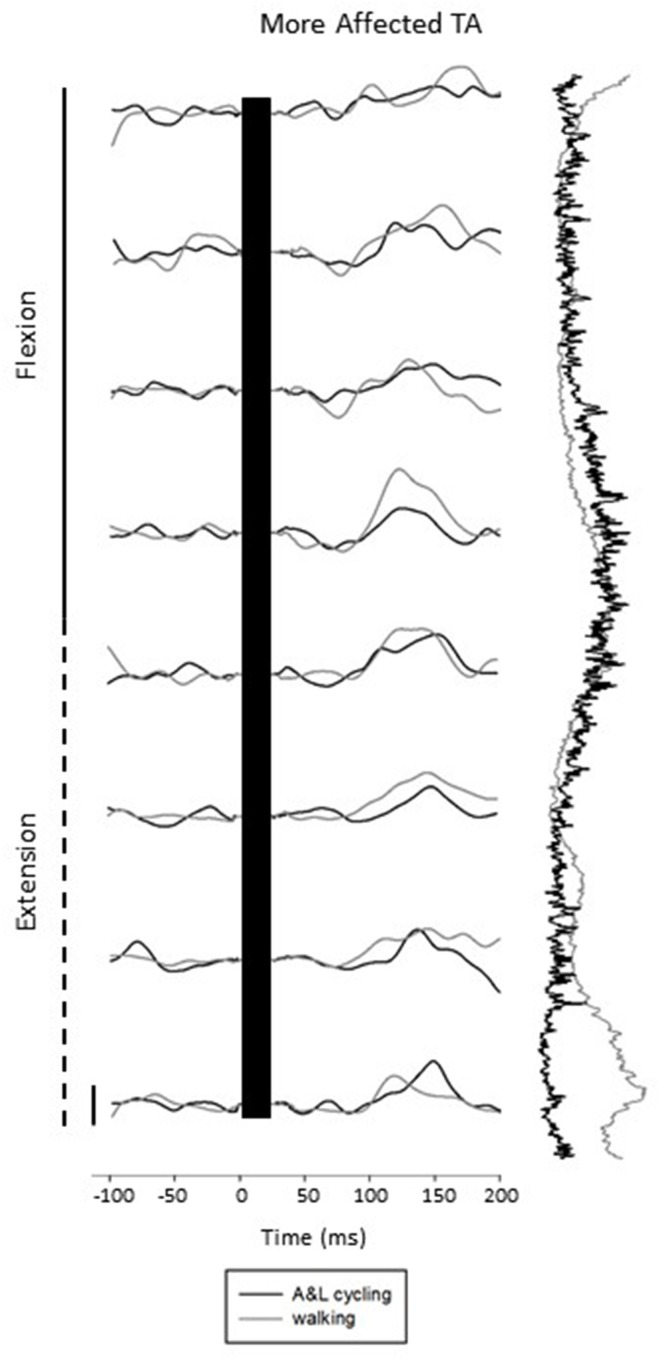
**Subtracted electromyographic (EMG) traces of the more affected tibialis anterior (TA) from a representative participant evoked by superficial radial and superficial peroneal nerve stimulation during A&L cycling and walking**. The stimulus artifact has been removed from each trace and replaced by a black bar extending from time 0 out to ~30 ms post stimulus. Background EMG during A&L cycling and walking is shown to the right of the trace plotted vertically. Calibration bar represents 10 μV.

Figure 3 shows subtracted EMG traces for A&L cycling (black line) and walking (gray line) for MA TA taken from one participant during SP+SR nerve stimulation. The figure displays subtracted EMG traces for each phase moving top to bottom from flexion to extension. To the right of the subtracted traces control EMG for A&L cycling (black line) and walking (gray line) is plotted vertically. Data in this figure visually illustrates similarities for cutaneous reflexes between A&L cycling and walking across 8 phases of movement.

**Figure 4:**
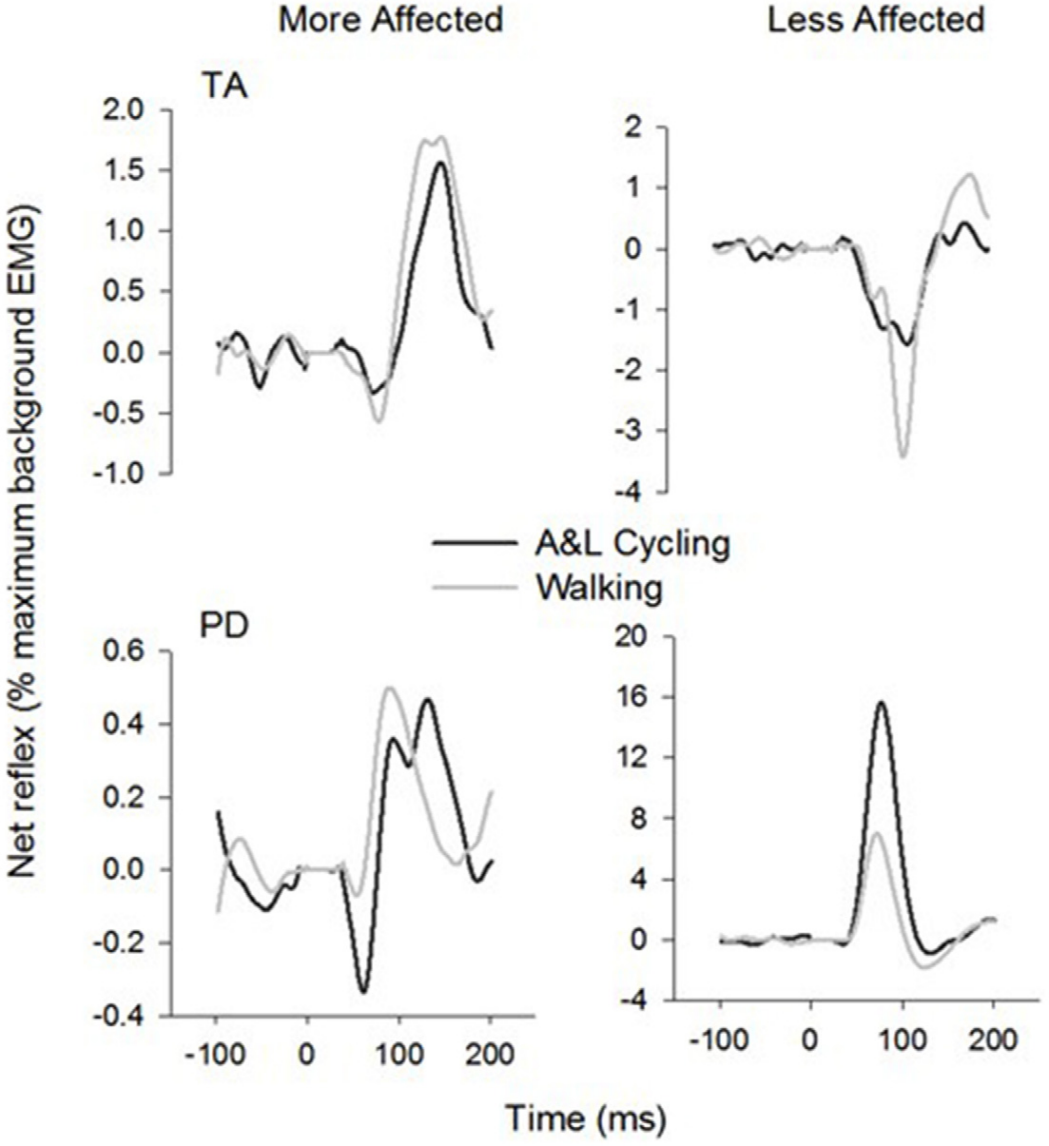
**Ensemble grand average subtracted reflex traces from all phases and all subjects of A&L cycling and walking**. There is a similar pattern of cutaneous reflex modulation across tasks. Note that despite some changes in amplitudes, the general pattern is conserved.

General conservation in the pattern of reflexes between tasks can be seen in Figure 4 where grand average reflex traces from SP+SR nerve stimulation during A&L cycling (black line) and walking (gray line) are plotted. Although some expected differences in amplitude were observed, general patterns of modulation (i.e., sign of response) are similar. Between tasks facilitation was seen bilaterally in the arms and seen in the MA leg while the in the LA leg suppression was observed.

**Figure 5:**
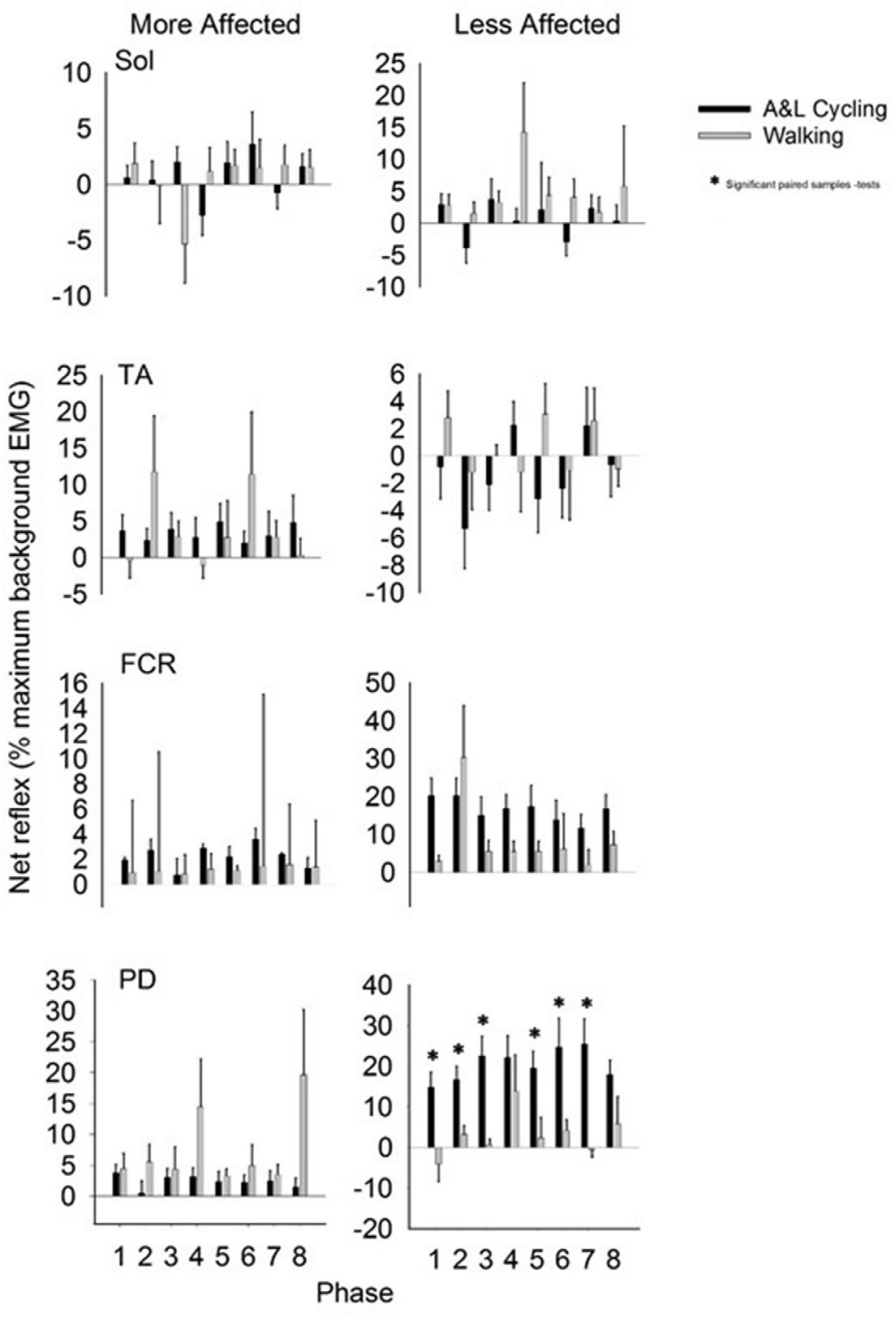
**Net cutaneous reflex from SP+SR stimulation for muscles of the more and less affected arm and leg averaged across all participants**. Black bars are for A&L cycling and gray bars are for walking tasks. Net cutaneous reflexes are means (± s.e.m) from all participants and are normalized to the peak control (i.e., background) EMG recorded in each task. Significant difference between tasks (^*^) were calculated with a paired samples *t*-test. Abbreviations are: Sol, soleus; TA, tibialis anterior; FCR, flexor carpi radialis; and PD, posterior deltoid.

Net reflexes evoked in the legs and arms following SP+SR nerve stimulation for all participants are plotted as bars in Figure 5. Values for A&L cycling (black bars) and walking (gray bars) are normalized and expressed as percentages of the peak background value for each phase for each participant. In the legs, there were no significant main effects of phase or task for any muscle and there were no interaction effects indicating that the pattern and amplitude of reflexes was similar between A&L cycling and walking. In the arms, there was a significant main effect of task in the MA PD and LA PD [*F*_(1, 10)_ = 7.267, *p* = 0.022, *d* = 0.781 and *F*_(1, 10)_ = 17.780, *p* = 0.002, *d* = 0.966]. While no significant differences in MA PD were detected by paired *t*-tests, there were significant differences for the LA PD between A&L cycling and walking across phases (see ^*^ in Figure 5).

Reflex amplitude is typically uncoupled from rhythmic background EMG amplitude in NI participants. To examine the extent to which reflex amplitudes were related to background EMG during A&L cycling and walking in stroke participants, we calculated Pearson's correlation coefficient. Across all eight muscles for each participant across all phases of A&L cycling and walking no significant correlations were found (see Table 1).

**Table 1:** **Correlation coefficients between the net reflex response and background EMG during A&L cycling and walking tasks**.

**Muscle**	**A&L cycling**	**Walking**
MA SOL	0.052	0.053
LA SOL	0.251	0.035
MA TA	0.097	0.153
LA TA	0.254	0.103
MA FCR	0.047	0.120
LA FCR	0.269	0.097
MA PD	0.051	0.141
LA PD	0.133	0.071

*The critical value for this 2-tailed comparison (p < 0.05) for 12 participants was 0.58. Abbreviations in text*.

### Mathematical PCA

**Figure 6:**
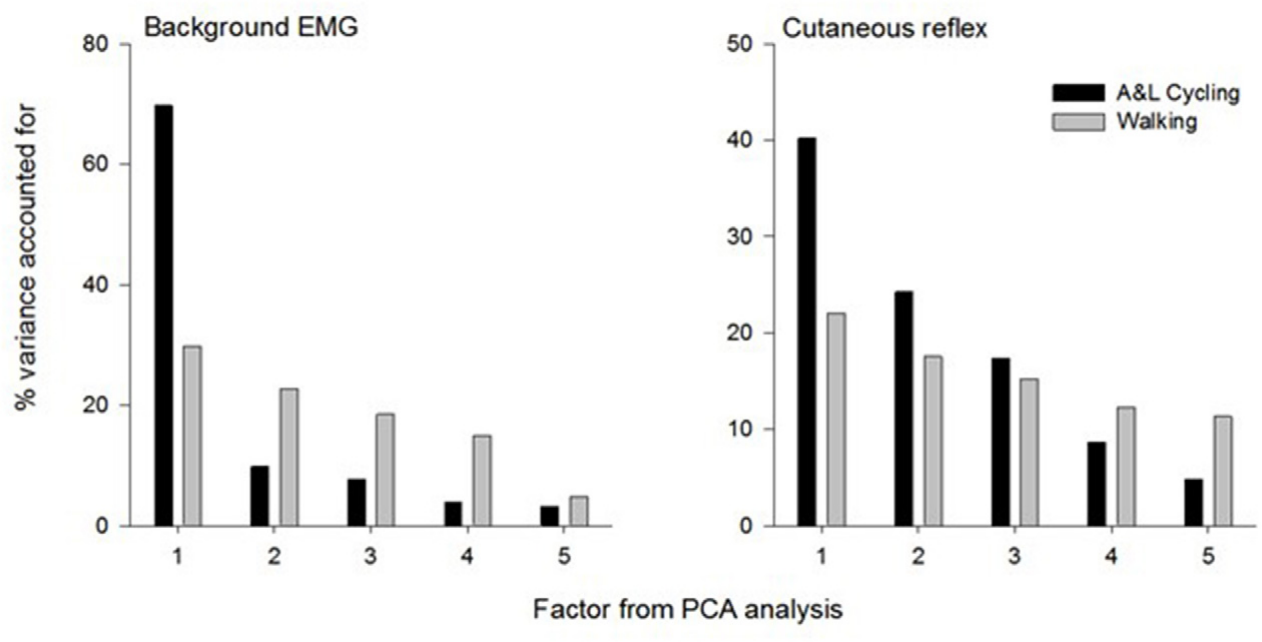
**Summary of principal component analysis for background EMG and cutaneous response in A&L cycling and walking**. Bars represent the variance accounted for by each factor.

The summary for the principal components analysis for combined SP+SR nerve stimulation in A&L cycling and walking is shown in Figure 6. The subplot on the left is for the variance accounted for (%VAF) from each principal component of background EMG amplitudes and the subplot on the right is for %VAF of cutaneous responses. Across both tasks, five common factors explained more than 86% of the variance for background EMG and 90% of the variance for the cutaneous response. There was a substantial difference between A&L cycling and walking in the magnitude of variance accounted for by the first principal component of background EMG and reflex modulation with 40–69% in cycling and only 22–29% in walking.

## Discussion

The purpose of this experiment was to evaluate the extent to which common neural regulation is conserved across locomotor tasks despite reduced supraspinal input after stroke. There were some but few significant differences between A&L cycling and walking for EMG amplitude modulation and net cutaneous reflex modulation (see Results) indicating that A&L cycling and walking have preserved modulation patterns after stroke. Some muscles displayed significant phase-dependent reflex modulation where no correlation to background EMG was present. Mathematical analysis revealed a dependence on five common factors explaining more than 86% of the variance for background EMG and 90% of the variance for the cutaneous reflex. These data suggest that after a stroke common neural patterning from conserved subcortical regulation in the arms and legs is conserved across locomotor tasks involving arm and leg movement. These results have translational implications for rehabilitation where A&L cycling could be usefully applied to recover walking function.

### The role of supraspinal input

When comparing the results from this study of mathematical extraction of variance to results from a similar study by Zehr et al. (2007) in NI participants, some differences can be noted. Firstly, in this study, more principal components are required to explain less variance. Here, five components are required whereas only four principal components are required to explain 93% of the variance across tasks in a NI population (Zehr et al., 2007). We suggest the additional components could reflect the reduced extent of supraspinal regulation following stroke. Secondly, a larger division in the %VAF by the first principal component is seen when comparing NI participants and stroke participants. When comparing between walking and A&L cycling in NI participants, the largest difference in %VAF for the first principal component is approximately 30% whereas for stroke participants the largest difference is approximately 45% (see Figure 6). Again, this indirect observation may be ascribed to reduced supraspinal input following a stroke rendering integrations between the arms and legs more complex without the fine-tuning provided by an intact supraspinal system.

Within the framework of comparing muscle synergies in NI and to those present after stroke, alterations in the number of modules extracted is often observed. This is likely attributable to altered spinal cord excitability interacting with changes in descending motor regulation. Impaired upper limb function is associated with decreased number of reflex synergies (Trumbower et al., 2010, 2013) and reductions in voluntary synergy structures during isometric tasks (Roh et al., 2011), when compared to NI participants. During walking, changes in the modular organization of muscle synergies is also demonstrated post-stroke where there is a reduction in the number of synergies extracted (Clark et al., 2010) and modified recruitment organization (Gizzi et al., 2011). Our results are similar in that the components extracted are changed compared to NI participants, however, here we report instead an increase in the number of principal components. Diminished cortical modulation consequent to a stroke lesion could cause deficits in muscle synergy coordination leading to the observed functional impairments in locomotion following a stroke. These may be characterized during locomotion as an increased number of principal components, each accounting for a lower %VAF than is found in the intact nervous system.

Conservation in nervous system control across task has been previously ascribed to the action of locomotor central pattern generators (CPG) modulating transmission in cutaneous pathways by premotoneual gating. However, differences between NI and stroke participants could arise from reduced supraspinal regulation of alphamotoneuronal and interneuronal activity caused by the stroke lesion (Dobkin, 2004, 2005). Descending supraspinal input can regulate reflex output through either modulation of excitability in the interneuron reflex pathways or through the internal networks that are part of the CPG itself (Zehr, 2005; McCrea and Rybak, 2008). Alterations in descending supraspinal regulation of interneuronal reflex pathways during rhythmic activity explains differences in neural conservation of locomotor tasks between stroke and NI participants. Some conservation of these mechanisms is still observed, thus implicating the spinal cord and subcortical areas in neural regulation across locomotor tasks.

Comparisons between the more and less affected limbs in stroke participants, can reveal the effects of reduced supraspinal input on reflex modulation. Responses in the tibialis anterior at approximately 80 ms were small or absent during walking in stroke participants, in those with hereditary spastic paraparesis and in those with a spinal cord injury (Jones and Yang, 1994; Zehr et al., 1998a; Duysens et al., 2004). Specifically, an absence of end-swing suppression in the TA (normally observed in NI participants) was noted however end-stance facilitations remained. This suggests that suppressions may be under the control of the cortex while facilitations are under the control of spinal CPGs (Duysens et al., 2004). In the data presented here (see Figure 4), this fits nicely as in the MA TA (influenced by the lesioned cortex but still under the control of spinal CPGs) mainly facilitations are present and in the LA TA mainly suppressions are present. Therefore an intact cortex and corticospinal tract are required for full expression of the full range of reflex modulation during locomotion.

### Evidence for conserved “common core”

Common control across rhythmic movement tasks could be the result of a common core of subcortical elements expressing neural activity to produce the basic pattern of arm and leg movement (Zehr et al., 1997, 2007; Zehr, 2005). That is, a central mechanism is likely responsible for regulating various types of rhythmic movement in a similar oscillatory fashion. Measurements of muscle activity across various rhythmic tasks have shown a consistent frequency relationship between arm and leg movements for walking, cycling, creeping and swimming which could be indicative of spinal interconnections between the upper and lower spinal CPGs that are engaged in the locomotor function (Wannier et al., 2001). Indeed, propriospinal linkages between the fore and hindlimbs have been identified in the cat (Lloyd, 1942; Gernandt and Megirian, 1961; Miller et al., 1973) and data on interlimb responses obtained in persons with cervical spinal cord injury (Calancie, 1991; Calancie et al., 1996) suggests that quadrupedal links between forelimb and hindlimb coordination are conserved in humans (Dietz et al., 2001; Wannier et al., 2001; Zehr et al., 2009).

The main results of this experiment demonstrate the persistence and modulation of reflexes during A&L cycling and walking after stroke despite the interruption of some descending regulation of interneuronal excitability arising from the supraspinal lesion. The overall similarities in modulation patterns for background EMG and cutaneous reflexes provide insight into the status of neural control circuits in the damaged nervous system (Zehr and Duysens, 2004). A contribution from subcortical and presumed spinal locomotor pattern generating networks is implicit in the observations here where networks for arm and leg coordination could reside in subcortical areas as damage to the brain following stroke does not seem to significantly affect common neural regulation (Zehr et al., 2004). These results add to existing evidence that portions of the neural circuitry regulating rhythmic arm and leg movements remain accessible and intact after stroke (Zehr and Duysens, 2004; Ferris et al., 2006; Zehr and Loadman, 2012; Zehr et al., 2012).

### Translational applications

The neural similarities between A&L cycling and walking observed here have translational implications for rehabilitation where A&L cycling could be usefully applied to recover walking function. This can be achieved by activation of a set of similar residual neural pathways to strengthen interlimb neuronal coupling to improve walking performance after stroke (Zehr, 2005; Ferris et al., 2006; Balter and Zehr, 2007; Zehr et al., 2007, 2009, 2012; Klimstra et al., 2009; Zehr and Loadman, 2012). In addition, A&L cycling is similar to walking in terms of muscle activity and joint ranges of motion (Zehr, 2005; Balter and Zehr, 2007).

Our experimental methods do not allow us to effectively delineate the specific locus of the observed reflex (intra- vs. interlimb) given we are using simultaneous stimulation of both the hand and foot (see Figure 5 in Nakajima et al., 2013). However, the presence of cutaneous reflexes seen here confirms that neuronal pathways linking the arms and the legs remain partially conserved in stroke providing a substrate for training induced plasticity to improve function. Combined arm and leg stimulation can be used as an index for arm and leg coupling where stimulation likely converges in reflex pathways from cutaneous inputs for the hand and foot to produce the responses observed (Nakajima et al., 2013). Cutaneous inputs and associated modulation of reflex amplitudes could serve as probes to monitor ensuing neuroplastic adaptations in interlimb pathways resulting from targeted rehabilitation (Wolpaw, 2010; Zehr and Loadman, 2012). In addition, the use of principal component analysis could provide a useful means of evaluating rehabilitation effects where reductions in the number of principal components and variance explained by each component could suggest improved control.

## Conclusion

In general, background locomotor EMG was similar between A&L cycling and walking where similar phase dependent modulation patterns were observed. Modulation of cutaneous reflexes from hand and foot stimulation suggest a conserved “common core” of subcortical regulation of locomotion despite altered descending supraspinal input from the stroke lesion. These results have translational implications for rehabilitation where A&L cycling could be usefully applied to improve walking function.

## Author contributions

Taryn Klarner, Trevor S. Barss, Pamela Loadman, Yao Sun, Chelsea Kaupp, and E. Paul Zehr contributed to the experimental design. Taryn Klarner, Trevor S. Barss, Yao Sun, and Chelsea Kaupp conducted the experiments. Taryn Klarner, Trevor S. Barss, Yao Sun, and Chelsea Kaupp participated in analysis of the data. Taryn Klarner and E. Paul Zehr wrote the paper but all authors commented on and approved the final draft of the MS.

### Conflict of interest statement

The authors declare that the research was conducted in the absence of any commercial or financial relationships that could be construed as a potential conflict of interest.
